# Bilateral Hypermetropia, Myelinated Retinal Nerve Fibers, and Amblyopia

**DOI:** 10.4103/0974-9233.75891

**Published:** 2011

**Authors:** Radha Shenoy, Alexander A. Bialasiewicz, B. Al Barwani

**Affiliations:** Department of Ophthalmology, Armed Forces Hospital, Muscat, Oman; 1 Department of Ophthalmology, Al Ahli Hospital, Doha, Qatar

**Keywords:** Amblyopia, Hypermetropia, Myelinated Retinal Nerve Fibers, Straatsma Syndrome

## Abstract

A 14-year-old hyperopic female with poor vision in both eyes was evaluated for ophthalmic and systemic diseases. The patient had bilateral retinal fiber myelination and greater vision loss in the more hyperopic eye. This was a rare case of reverse Straatsma syndrome, the clinical presentation which may be accompanied with significant vision loss.

## INTRODUCTION

Myelinated retinal nerve fibers have been referred to as “papillae leporina” and are often detected incidentally as isolated asymptomatic white grey lesions obscuring retinal details. A significant visual loss associated with myelinated nerve fibers is uncommon. However, amblyopia associated with myopia and myelinated nerve fibers is called Straatsma syndrome.

In this case report we document a case of reverse Straatsma syndrome in a young female patient exhibiting bilateral asymmetric hypermetropia, and myelinated nerve fibers, which were more extensive in the amblyopic eye with the greater refractive error.

## CASE REPORT

**Figure 1:**
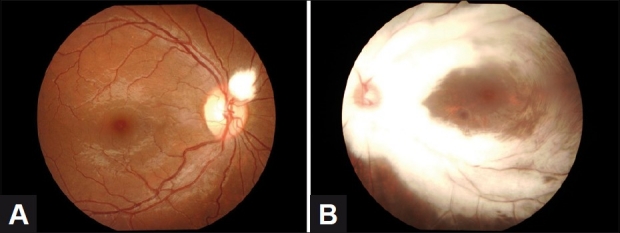
(A) Right eye showing a tuft of myelinated fiber at upper nasal pole of the optic disc. (B) Left eye showing peripapillary myelination with extension along arcades. Posterior pole is not myelinated

**Figure 2:**
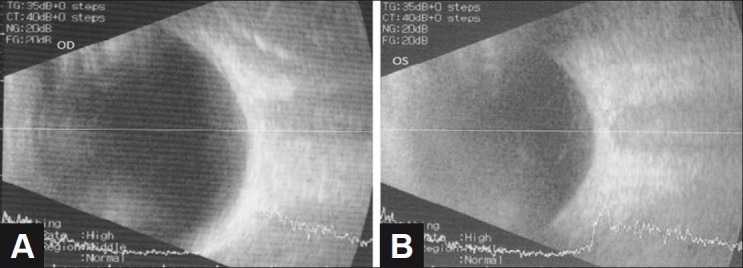
(A,B) Axial length of the right eye and the left eye

A 14-year-old female was referred from school health services for poor vision. She underwent a detailed ophthalmic and systemic examination. The visual acuity was 6/6 (OD) with +1.0 sphere, and 6/36 (OS) with +3.0 sphere. Slit-lamp examination revealed normal anterior segments. Intraocular pressure (Goldmann applanation tonometer) was 12 mmHg bilaterally. Myelinated fibers were present in both eyes. In the right eye, the cup to disc ratio (CDR) was 0.3, there was a tuft of myelinated nerve fiber at a distance of about 1/4 to 1/3 disc diameter (DD) at the superior nasal pole of the disc obscuring the beginning of the superior nasal artery and vein [Figure 1A]. The blood vessels and fovea were normal. In the left eye, the CDR was 0.3; there was peripapillary myelination that extended from the two poles of the disc along the arcades [Figure 1B]. The macula and nasal retina appeared free of myelination. A-scan biometry revealed an axial length of 23 mm OD and 21 mm OS [Figures 2A, B]. Systemic examination for tumors and inflammations was unremarkable.

## DISCUSSION

In a series for 3968 autopsies (7936 eyes), Straatsma and colleagues noted that retinal myelination occurred in 0.54% of the eyes. In the cases with retinal myelination, Straatsma found 0.98% of the cases were unilateral and 7.7% were bilateral.^1^ In 33%, the myelination was contiguous with the optic nerve head.^1^ Four patients had a typical presentation characterized by ipsilateral extensive myelinated retinal nerve fibers, anisometropic myopia, amblyopia, and strabismus.^1^ Glial cells and concentric lipoprotein lamellae that formed the myelin sheath, but morphologically normal sensory retina was noted in all eyes, under light microscopy and electron microscopy.^1^ Three different types of myelination have been described-type 1 occurring along the superior temporal arcade, type 2 along both the arcades and type 3 with no contiguity with the disc.^2^ According to this classification, our patient had type 2 myelination in the left eye.^3^

Myelinated retinal nerve fibers are more common in myopic than in hypermetropic eyes.^3^–^5^ In patients with bilateral hypermetropia, myelinated retinal nerve fibers occur in the eye with lower refractive error.^3^ Axial myopia is reported in 35-58% of patients with myelinated nerve fibers in previous studies, with 83% showing myopia of greater than 6 D. Amblyopia, (high) myopia, and myelinated nerve fibers form a syndromic triad (“Straatsma’s syndrome”) with a prevalence of 0.03-10%, amblyopia may be functional, anisometropic or organic due to associated macular or ocular anomalies and strabismus may or may not be present.^3^,^4^,^6^

The cause of retinal fiber myelination remains unknown. Some believe it causes myopia and others theorize that it is the result of myopia.^5^ Blurring of retinal image inducing visual deprivation, at a critical stage of ocular development causes axial enlargement of the eye and development of myopia, which in turn delays development of the lamina cribrosa, thereby allowing extension of myelination in the retina.^1^,^4^,^6^,^7^ Our patient was a bilateral hyperope with bilateral retinal myelination, which was extensive in the eye with shorter axial length and the higher refractive error. This case contradicts the former hypothesis, excluding the latter and supporting the theory of anomalous distribution of oligodendrocytes as an etiologic factor.^1^,^3^–^7^

Scotomas in myelinated retinas are smaller than predicted by the extent of myelination, suggesting that light penetrates to the photoreceptor layer despite the myelin interference.^1^,^6^,^7^ In the absence of other structural ocular abnormalities and presuming a morphologically normal sensory retina, the amblyopia in our patient probably is likely due to the anisometropia. A refractive difference of 1.25 D or greater in hyperopes is generally required for the development of amblyopia, unlike myopes, where an even greater difference seems to be required.^3^–^5^ Consistent with this observation, our patient had bilateral hypermetropia and anisometropia of +2.0 D.

The presence of bilateral myelinated nerve fibers in hypermetropes is rare. Amblyopia with extensive myelination of the retinal nerve fibers, particularly in the eye with greater hypermetropia, may refute the existing relationship between myelinated nerve fibers and myopia.^1^–^7^ This clinical entity is called “reverse” Straatsma’s syndrome.^5^ Anisometropia seems to have a stronger influence on the relative visual acuity of each patient’s eyes than the presence of retinal nerve fiber myelination as illustrated in this case report.^1^–^7^
